# Toxoplasmosis Screening during Pregnancy in a Romanian Infectious Diseases Tertiary Center: Results of a 15 Years Follow-Up Program

**DOI:** 10.3390/microorganisms11092189

**Published:** 2023-08-30

**Authors:** Violeta Briciu, Angela Monica Ionică, Mirela Flonta, Ariana Almaș, Monica Muntean, Adriana Topan, Melinda Horvat, Liviu Ungureanu, Mihaela Lupșe

**Affiliations:** 1 Department of Infectious Diseases, “Iuliu Hațieganu” University of Medicine and Pharmacy, 400348 Cluj-Napoca, Romania; 2 Clinical Hospital of Infectious Diseases of Cluj-Napoca, 400348 Cluj-Napoca, Romania

**Keywords:** *Toxoplasma gondii*, antenatal screening, anti-*Toxoplasma* antibodies, acute *Toxoplasma* infection

## Abstract

Maternal infection with *Toxoplasma gondii* during pregnancy may have serious consequences for the fetus. In Romania, screening for toxoplasmosis is included in the first antenatal visit. A retrospective study was performed on all toxoplasmosis antenatal screening patients between May 2008 and February 2023. Twenty-seven thousand one hundred sixty-nine (27,169) pregnant women presented for prenatal screening once (22,858) or several times: during the same pregnancy (209) or during multiple pregnancies (4102). Thirty-one thousand six hundred fifty-eight (31,658) tests for IgM and IgG antibodies were performed. Nine thousand eighty-three (9083) tests (28.69%), corresponding to 7911 women (29.12%), were positive for IgG antibodies. The seroprevalence increased with patients’ age, decreased in time intervals, and was more frequently associated with rural residence. At risk for acquiring the infection during the pregnancy were women with negative anti-*Toxoplasma* IgG antibodies (70.88%), but only 0.9% of them presented for rescreening during the same pregnancy. Acute *Toxoplasma* infection (ATI) was suspected in 44 patients (0.16%) due to IgG seroconversion and/or low or borderline IgG avidity. A questionnaire follow-up interview was performed, and no congenital toxoplasmosis was identified in children born from mothers with probable ATI. Our study demonstrates poor compliance with the screening program in the Romanian population.

## 1. Introduction

*Toxoplasma gondii* is a coccidian parasite that localizes in the small intestine of felines, which act as final hosts and reservoirs [1]. The life cycle is indirect and can involve numerous species of birds and mammals, including humans, as intermediate hosts, in which the parasite becomes encysted in the muscles and various organs [2]. Humans may acquire infection by ingesting infective elements, such as tissue cysts in raw or undercooked meat or oocysts shed by infected cats, by ingesting contaminated food (fruits, vegetables) and water [1,3]. *T. gondii* is amongst the most studied parasites worldwide, having the fourth position in the global ranking of food-borne parasites [4] and second in Europe, based on a multicriteria ranking tool for scoring [5]. A recent publication that evaluated 152 published studies determined an overall range of seroprevalence in 648,010 subjects between 0.5–87.7%, with an average global seroprevalence rate of 25.7%. The African countries had the highest average seroprevalence rate of 61.4%, followed by Oceania (38.5%), South America (31.2%), Europe (29.6%), USA/Canada (17.5%), and Asia with 16.4% [6].

Innate and adaptive immune responses to *T. gondii* are being extensively studied, as reviewed by [7], while few studies have addressed the immune response to toxoplasmosis in pregnancy [8]. Maternal infection with *T. gondii* during pregnancy may have serious consequences for the fetus, ranging from miscarriage, severe symptoms including cerebral calcification, hydrocephalus or microcephaly, seizures, developmental delays, chorioretinitis, strabismus, vision loss, hearing loss, hepatosplenomegaly, jaundice, petechiae, thrombocytopenia, anemia and/or transaminitis [9]. A study in Italy reported the incidence of seroconversion and suspected infection during pregnancy at 0.8 per 1000 live births and congenital toxoplasmosis at 0.1 per 1000 live births [10].

Infection in pregnant women is usually asymptomatic, so the diagnosis relies on serological tests. Systematic screening programs for all pregnant women at risk are not widely implemented, and there is no consensus about serological screening for *T. gondii* IgG and IgM antibodies in pregnant women. It is not recommended by the American Academy of Pediatrics in the United States [11] or Canada [12]. Based on the last National Screening Committee review of this condition in the UK, released in August 2016, screening for toxoplasmosis in pregnant women is not currently recommended [13]. Five countries officially recommend mandatory prenatal screening in Europe, either by monthly testing (France, Italy) or by three-monthly testing of susceptible women (Austria, Lithuania, Slovenia) [14]. In France, screening for toxoplasmosis in pregnancy has been performed since 1992, with serological testing at the first-trimester prenatal visit, with monthly serologic follow-up until delivery of pregnant women identified as “not protected against toxoplasmosis” [15], but late presentation and poor adherence to screening was described in practice [16]. In Austria, *Toxoplasma* screening of pregnant women is covered by national healthcare providers and The Ministry of Health, with a recently published cost-effectiveness analysis but dealing with the same poor adherence to the screening scheme [17,18,19]. In most other European countries, there is no recommendation or a formal recommendation not to screen. In Romania, screening for toxoplasmosis is included in the first antenatal visit as recommended by the Health Ministry in “The protocol regarding the methodology of prenatal and postnatal consultation documented in the pregnant woman’s book” and reimbursed by the National Health Insurance System, with no further official recommendation on retesting protocol [20].

The global IgM and IgG seroprevalence in pregnant women ranges between 1.9 and 32.9%, with statistically significant differences between WHO regions [21]. Regarding acute *Toxoplasma* infection (ATI) during pregnancy, a systematic review and meta-analysis of 217 studies comprising 902,228 pregnant women across 74 countries identified an overall prevalence of 1.1% [22]. The diagnosis of ATI during pregnancy can be made based on the detection of low avidity serum anti-*Toxoplasma* IgG antibodies or seroconversion from IgG negative to IgG positive status (usually combined with detection of anti-*Toxoplasma* IgM antibodies) in cases of sequential testing during pregnancy. The IgG avidity test, which measures the affinity of IgG antibody binding to *T. gondii* antigens, is low during the acute stages of infection and becomes high as the infection progresses to the chronic stage. Low IgG avidity may distinguish between *T. gondii* infection acquired <12–16 weeks vs. >12–16 weeks from testing and is particularly useful for differentiating acute from chronic maternal infections early in gestation [23].

Globally, the annual incidence of congenital toxoplasmosis is estimated to be 190,100 cases (179,300–206,300) (accounting for 1.2 million disability-adjusted life years (DALYs) annually), the global incidence of congenital toxoplasmosis was 1.5 per 1000 livebirths, with high burdens seen in South America, Middle Eastern and low-income countries [3].

The main objectives of our study were to investigate the prevalence of ATI in pregnant women screened for toxoplasmosis by serology, to perform a follow-up of pregnancies’ evolution and congenital *Toxoplasma* infections, and to provide data on seroprevalence of specific anti-*Toxoplasma* IgG antibodies in investigated pregnant women from Northwestern Romania. The secondary objectives were to evaluate possible risk factors associated with *T. gondii* infection and the evolution trend of seroprevalence over fifteen years.

## 2. Materials and Methods

### 2.1. Study Design and Setting

A retrospective study on all consecutive investigated female patients for antenatal screening of anti-*Toxoplasma* antibodies was performed in The Clinical Hospital of Infectious Diseases, a large Tertiary Center located in Cluj County, North-Western Romania.

The study was approved by the Ethics Committee of the Clinical Hospital of Infectious Diseases Cluj-Napoca through decision 8268/11.04.2023.

Evaluation of TORCH (*Toxoplasma gondii*, *Rubella* and *Cytomegalovirus*) serology has been performed in the hospital ambulatory starting from 2008; after evaluation of serology results, recommendations and counselling are provided to pregnant women, based on the local protocol. The local protocol recommends screening for *T. gondii* in the first trimester of pregnancy and retesting in seronegative women in the second and third trimesters. Amniocentesis is recommended after the 18th week of pregnancy, and PCR for *T. gondii* in the amniotic fluid in probable ATI (documented IgG seroconversion or IgG low/borderline avidity in case of both IgM and IgG *Toxoplasma* positive results). Treatment is recommended in ATI to prevent mother-to-child transmission, with spiramycin in the first trimester, switched to pyrimethamine+sulphadiazine+folinic acid from the second trimester of pregnancy in case of positive PCR result in amniotic fluid, or no amniocentesis performed. In case of PCR negative result, spiramycin should be continued until birth. Counseling for keeping the *Toxoplasma* seronegative status during pregnancy is also provided.

### 2.2. Participants

All pregnant women who presented between 14 May 2008 and the 2 February 2023 were retrospectively included by accessing the hospital electronic database. Their identification was possible based on identifying the protocol dedicated to TORCH protocol in pregnant women, using the coding proposed by the International Statistical Classification of Diseases and Related Health Problems 10th Revision (code Z36.9). For patients identified with probable ATI (documented IgG seroconversion or IgG low/borderline avidity in case of both IgM and IgG positive results), a cross-sectional evaluation was performed by a telephone follow-up, using a consent form and a questionnaire (Supplementary Materials).

### 2.3. Variables

Data collected were age, date of investigation, demographic data (urban/rural residency, county), results of IgM and IgG anti-*Toxoplasma* antibodies, and avidity of IgG anti-*Toxoplasma* antibodies. Multiple serial results were recorded for the same patient, performed during one pregnancy or consecutive multiple pregnancies. The telephone questionnaire was performed by one of the study investigators in April–May 2023. Questions addressed the evolution of pregnancy (normal evolution to birth, miscarriage, or stillbirth), diagnostic investigations performed (amniocentesis), treatment during pregnancy for toxoplasmosis, and congenital toxoplasmosis diagnosed in the child or manifestations that could be associated with congenital toxoplasmosis.

### 2.4. Laboratory Assays

The anti-*Toxoplasma* gondii antibody screening was performed using commercially available enzyme-linked immunosorbent assay (ELISA) kits (ImmunoLISA Toxo IgM Capture, NovaTec Immundiagnostica, Dietzenbach, Germany; ENZYWELL TOXOPLASMA IgG, DIESSE Diagnostica Senese, Monteriggioni, Italy; Anti-*Toxoplasma gondii* ELISA (IgM, IgG), EUROIMMUN, Lübeck, Germany; Serion Elisa *Toxoplasma Gondii* IgM, IgG, SERION Diagnostics, Würzburg, Germany), or automated anlyzers (VIDAS, a two-step enzyme immunoassay sandwich method with a final fluorescent detection (ELFA), BIOMÉRIEUX, Craponne, France; or ARCHITECT i1000SR, later replaced by Alinity: fully automated, 2-step chemiluminescent microparticle immunoassay (CMIA), Abbot Laboratories, Chicago, IL, USA), according to availability of kits and consumables.

The IgG avidity tests were performed using an automated analyzer (VIDAS, BIOMÉRIEUX, Marcy-l’Étoile, France), and the attained values were classified as low (values below 0.2), borderline (values between 0.2 and 0.3), or high (over 0.3).

### 2.5. Statistical Analysis

The statistical analysis was performed using EpiInfo^TM^ 7.2 software (CDC, Atlanta, GA, USA). The prevalence and 95% Confidence Interval (CI) were calculated globally and according to specific groups. The differences in prevalence were assessed using the chi-squared test and regarded as significant at *p* < 0.05. In the case of women with multiple presentations, the first positive test was considered for further calculations, or the first performed test in the case of constantly negative patients. The evolution diagrams in case of multiple presentations were drawn using a freely available online tool [24].

## 3. Results

During the study period, 27,169 pregnant women presented for prenatal screening once (22,858 women) or several times: during the same pregnancy (209 women) or during multiple pregnancies (4102 women). The demographic characteristics are presented in Table 1.

**Figure 1:**
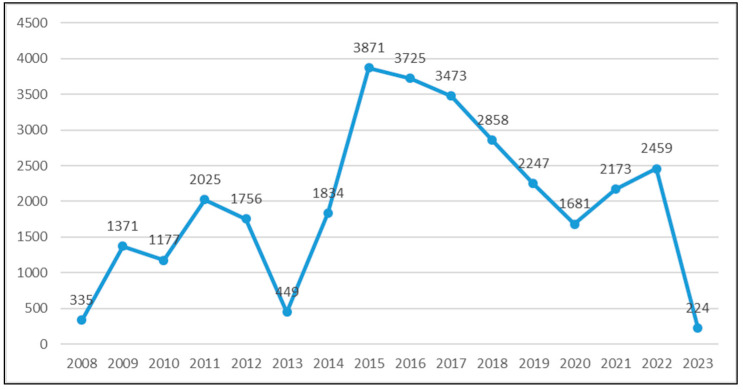
The annual distribution of anti-*Toxoplasma* IgM and IgG antibody tests.

Out of 31,913 serological tests performed for anti-*Toxoplasma gondii* antibodies, 31,658 tests included both IgM and IgG and were used for further calculations (the excluded tests were: six tests performed for IgG avidity only, 108 tests performed only for IgM, and 141 tests only for IgG). The annual distribution of tests performed is represented in Figure 1.

Overall, 9083 tests (28.69%) were positive for IgG anti-*Toxoplasma* antibodies (Table 2). The proportion of IgG-positive tests increased with age, and differences among age groups were statistically significant (χ^2^ = 79.99; d.f. = 8; *p* < 0.0001).

The seroprevalence was significantly higher (χ^2^ = 128.6; d.f. = 1; *p* < 0.0001) in women having a rural residence (6641; 34.4%), as compared to those living in the urban environment (2163; 27.5%).

The seroprevalence in IgG anti-*Toxoplasma* antibodies decreased in five-year intervals during the study period, a statistically significant decrease, as presented in Table 3.

A total of 324 tests (1.02%) were positive for IgM antibodies (Table 2). There were no statistically significant differences between age groups (χ^2^ = 1.69; d.f. = 8; *p* = 0.989), nor between rural (178; 0.92%) and urban (84; 1.07%) residents (χ^2^ = 1.29; d.f. = 1; *p* = 0.243).

In total, 22,251 tests (70.29%; 95% CI 69.78–70.79) were negative for both IgM and IgG antibodies (Table 4). On the tests that were positive for IgM, no seasonality was observed: 4.63% were detected in December, and 12.04% were detected in January, and the distribution of cases during the other months ranged between these values and was not significantly different.

### 3.1. Patients with Single Presentation for Screening

**Figure 2:**
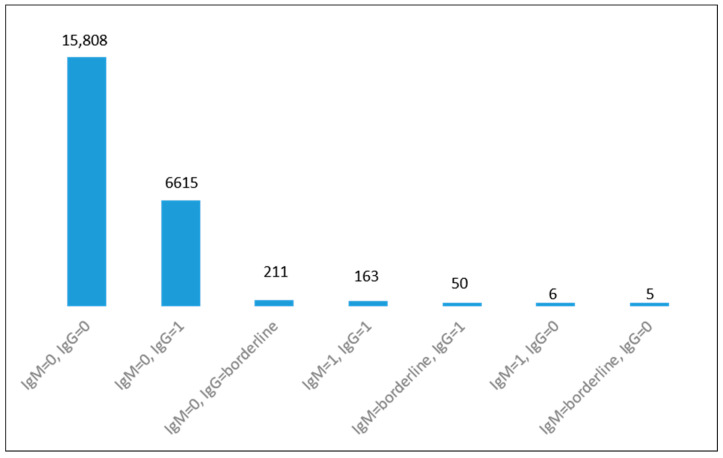
Test serological results for patients who presented once for screening: 0 = negative; 1 = positive.

For patients having a single presentation, the serological results for IgM and IgG antibodies are detailed in Figure 2. Of the 22,858 patients who presented only once for testing, 15,808 patients had both IgM and IgG negative results, 211 IgM negative and IgG borderline. Six patients had IgM positive and IgG negative; five had IgM borderline and IgG negative. In total, 16,030 patients (70.13%) were at risk for ATI during the pregnancy, but did not present for retesting in our medical unit.

### 3.2. Serological Profiles during the Same Pregnancy

**Figure 3:**
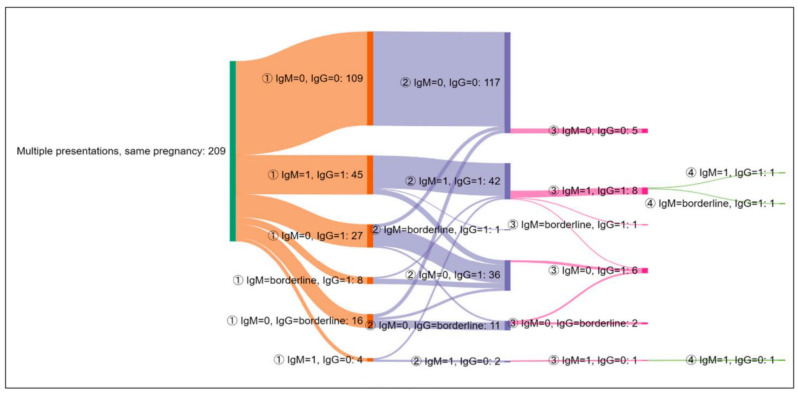
The dynamics of test results in women with multiple presentations during the same pregnancy. The number of patients is presented next to each serological profile. 0 = negative; 1 = positive. The circled numbers represent the number of the screening test (from the first up to the fourth).

The serological profile dynamics for the 209 women investigated more than once during the same pregnancy are presented in Figure 3.

One hundred and eighty-six (186) patients presented only two screening serological evaluations for *T. gondii* during the same pregnancy.

Between the first two tests, the levels of IgG antibodies increased in six women as follows: from negative to positive in two patients and from borderline to positive in four patients.

A total of 20 pregnant women were tested three times during the same pregnancy. Between the second and third tests, the IgG levels increased from borderline to positive in two women. No seronconversion from negative to positive IgG was documented.

Three patients had four tests performed. In one patient, a persistent IgM serological profile was described, with no seroconversion for IgG, interpreted as false positive IgM.

### 3.3. Serological Profiles during Multiple Pregnancies

**Figure 4:**
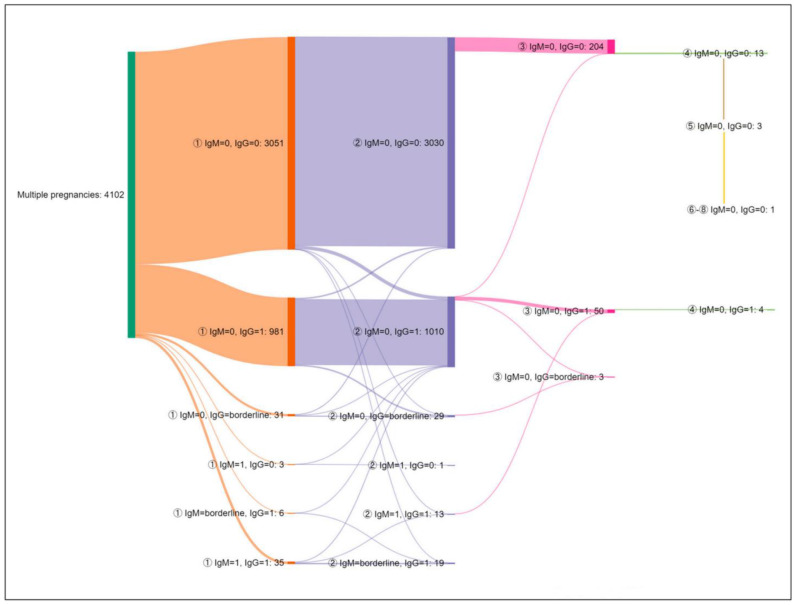
The dynamics of test results in women with multiple pregnancies. The number of patients is presented next to each serological profile. 0 = negative; 1 = positive. The circled numbers represent the number of the pregnancy (from the first up to the eighth).

The serological profile dynamics of multiple pregnancies for 4102 patients is presented in Figure 4.

Between the first and second pregnancy, the levels of IgG increased in 62 women as follows:

- from negative to positive in 48 women who were initially negative for both IgM and IgG,
- from negative to positive in two patients who had IgM positive during the first pregnancy
- from negative to borderline in one woman who was initially negative for both IgM and IgG
- from borderline to positive in 11 patients.

IgG results changed from positive to negative in 21 patients and from positive to borderline in 13. Positivity for both IgM and IgG during the second pregnancy was noted for 13 women, of which 11 had the same profile during the first pregnancy, while two were initially negative.

From the second to the third pregnancy, there was no seroconversion from IgG negative to IgG positive in the 244 tested patients. IgG results changed from positive to negative in two patients and positive to borderline in one patient.

Of the 14 women investigated in four pregnancies, four were constantly positive for IgG, and the rest maintained negativity. Two women had five pregnancies, while one had eight. All three were constantly negative for both IgM and IgG.

### 3.4. Avidity Tests

In total, 186 avidity tests were performed for 118 patients, of which 79 had high values, 21 had low avidity, and 17 had borderline values.

### 3.5. Acute Toxoplasma Infection in Pregnancy

At risk for acquiring the infection during pregnancy were women with negative anti-*Toxoplasma* IgG antibodies (19,258 out of 27,169 women; 70.88%, in the present study). Only 174 patients at risk, representing 0.9% (174/19,258), returned for rescreening during the same pregnancy in our medical service.

Through repeated screening, we have confirmed seroconverion between the first two screenings in six patients out of the 186 who were tested twice and in two additional patients from the 20 who retested three times. Overall, we have confirmed seroconversion in eight patients out of 209 who were retested (3.83%), two being investigated and confirmed with low IgG avidity.

By avidity testing, we confirmed ATI in 38 patients with IgM positive and IgG positive/borderline and low avidity (21 patients) or borderline avidity (17 patients). ATI was suspected in 44 out of the 27,169 pregnant women who presented for prenatal screening during the study interval (0.16%).

### 3.6. Mother-to-Child Transmission

We have tried to contact all the 44 patients classified as ATI. The telephone number for contact was not found in 16 patients. Of the other 28 patients, 12 patients consented to respond to the questionnaire. The diagnosis was established early in the pregnancy in all cases: at 12 weeks in two cases, before the eighth week for four patients, while the others declared it was during the third month, without mentioning the week. Amniocentesis was performed in four patients (but *Toxoplasma* PCR result could not be verified), and treatment with spiramycin was followed by six patients, while no pyrimethamine + sulphadiazine and folinic acid treatment was recorded. Of the 12 interviewed patients, one decided to have an abortion, while the rest gave birth to healthy newborns. Four children were serologically tested during their first year, with negative results. The questions addressed did not identify any health problems that could be interpreted as suspicion of congenital toxoplasmosis in undiagnosed children.

## 4. Discussion

Our study presents an important number of serological screenings performed for *T. gondii* infection in pregnant women in a tertiary infectious diseases’ hospital during a 15-year interval. Based on the number of 27,167 pregnant women investigated, 31,658 tests performed with both IgM and IgG anti-*Toxoplasma* antibodies, and 187 IgG avidity tests, as far as we know, this is the largest serological study on *T. gondii* serology in pregnant women from Romania so far. Testing for *Toxoplasma* continued even during the COVID-19 pandemic, 2020–2022, despite the lockdown, and the Clinical Hospital of Infectious Diseases of Cluj-Napoca was a first-line COVID-19 dedicated hospital. Investigations for pregnant women continued in a separate ambulatory building to reduce the risk of hospital-acquired SARS-CoV-2 infection.

### 4.1. Seroprevalence of Anti-Toxoplasma IgG

In our study, the overall positivity of IgG antibodies in performed tests was 28.69% and declined over time. As per women tested, the total seroprevalence was 29.12% (7911 of 27,169), showing a significant decrease in time (Table 2).

The largest study published until now from the western part of Romania on pregnant women screened for *T. gondii* infection (6889 patients) found a seroprevalence of specific IgG anti-*Toxoplasma* antibodies of 43.79% (on 1457 participants, between 2008–2010), compared with 38.81% in the second tested period (2015–2018, 5432 participants) with a decrease in time [25]. Similarly, in the United States, the age-adjusted seroprevalence of *T. gondii* among women of childbearing age (15–44 years) has declined over time (15%, 11%, and 9% in 1988–1994, 1999–2004, and 2009–2010, respectively) [11].

All countries running screening programs report a rise in the proportion of pregnant women who are seronegative [13]. This continuous decline in seroprevalence is likely associated with the lower parasite presence within the meat, strengthening hygiene measures in the food industry, decreased exposure to oocysts, better hygiene, and increased awareness in the population [26].

The IgG seroprevalence was significantly higher in women having a rural residence than those living in the urban environment. Though with higher seroprevalences than in our study, similar differences in results were found in a study on pregnant women in Western Romania [27]. This may be explained by differences concerning human eating habits and the presence of cats or gardening and contact with oocysts in the soil around the house, which could be an important risk factor in this rural context [28].

The age group distribution in investigated patients presents even extreme ages for pregnancies, as 12 patients were recorded in the age group 13–15 years old. The proportion of IgG-positive women increased with age, up to 46.43% in the age group 45–50 years old, and differences in prevalence among age groups were statistically significant. An increase in seroprevalence with an increase in age is also reported in women of reproductive age in Romania [27,29] and in blood donors from Romania [30]. The relationship between seroprevalence and age is frequently observed and reflects that the risk of toxoplasmosis infection remains throughout life.

*Toxoplasma gondii* infection or disease rates may be higher in some racial or ethnic groups, as has been shown in the general US population [31]; similarly, among HIV-associated hospitalizations, those for black persons were significantly more likely to be toxoplasmosis-related than for white persons, while among non–HIV-associated hospitalizations, persons of Hispanic ethnicity, Asian/Pacific Islanders, were more likely to have toxoplasmosis-related hospitalizations [32]. However, as the majority of the population in Romania is Caucasian [33], no analysis of race or ethnic group was performed in our study. The prevalence of obesity has reached alarming levels in Romania, where there is an estimated prevalence of 16.9% [34], but no association between IgG *Toxoplasma gondii* and overweight was found in a multinational epidemiological study that investigated the association between 7 serological markers of infections and body mass index [35].

### 4.2. Rescreening for Seroconversion in Toxoplasmosis Seronegative Women

A high percentage of women in our study were at risk for ATI during pregnancy (70.88%), as no IgG antibodies were detected. All were counselled by an infectious diseases specialist on reducing the risk of infection during the pregnancy, in accordance with international recommendations [36] included in the local protocol. However, all were recommended to return for retesting in the next trimester of pregnancy, and only a low percentage presented for rescreening during the same pregnancy. That could be explained by possibly retesting in another medical service or, most probably, poor compliance with the screening program. Studies from Austria, France and Italy have shown poor adherence to the screening scheme for maternal *T. gondii* infections in pregnancy, demonstrated by the fact that many recommended examinations are missed. In France, only 40 percent of pregnant women are tested seven or more times as recommended [16], and in Austria, “blind periods” in screening testing are reported [19]. The concerns about the adherence to screening policies with multiple antenatal appointments and tests are included in the UK Screening Committee review that recommended in 2015 against antenatal screening for toxoplasmosis [13]. The American Academy of Pediatrics recommended against implementing the French screening model for pregnant women in the USA, as it is unrealistic in the current American healthcare system and argued for a possible low adherence rate in pregnant women in the United States [11].

### 4.3. Acute Toxoplasma Infection in Pregnancy

In our 15 years retrospective study, by screening for ATI in 27,169 pregnant women, we have identified 44 patients (0.16%) with possible ATI based on IgG seroconversion and/or both IgM and IgG positive results with low or borderline IgG avidity test. Although detection of both IgM and IgG specific anti-*T. gondii* antibodies in a single serum sample may suggest an acute infection, a past infection cannot be excluded because IgM antibodies persist for months or years after infection [15]. Specific IgG avidity testing can help discriminate between these two possibilities. A high avidity result obtained on a first-trimester serum sample indicates a past infection, which occurred before pregnancy, whereas a low or intermediate avidity result is not informative in dating the infection [37]. Given this limitation, we have classified patients with low or borderline IgG avidity results as possible ATI.

The low adherence to the screening program might explain the low percentage of identified possible ATI (0.16%), as only 174 out of 19,258 IgG seronegative patients (0.9%) presented for rescreening. A recently published systematic review and meta-analysis of 217 studies identified an overall prevalence of ATI in pregnant women of 1.1% [22]. A limitation in the interpretation and comparison of results might be the low number of pregnant women who repeated screening.

We have even identified patients with IgM positive and IgG negative results (six patients) or IgM borderline and IgG negative results (five patients) who did not have a follow-up serology performed. This data might also suggest poor compliance with the screening program.

UK National Screening Committee underline that there is also a risk that women with negative IgM and IgG *Toxoplasma* serology are incorrectly told that they have a low risk of infection and do not present for interpretation of laboratory tests and further recommendations [13,36], but all our patients were counselled and received written recommendations for retesting.

Though intended for collection, we have not succeeded in finding in the electronic files of the patients information of pregnancy week at testing, so we have no data regarding compliance to early presentation for screening. Late initial testing was common in countries like France, with a long experience testing for toxoplasmosis in pregnant women (25% of French pregnant women had their first test performed late) [16].

A study in Northern Italy looked at adherence to screening among Italian and migrant women. After 12 weeks of gestation, late screening was recorded in 13.6% of Italian women and 31.9% of immigrant women [38].

### 4.4. Mother-to-Child Toxoplasmosis Transmission

Our evaluation by interview in all identified probable ATI in pregnant women tried to assess mother-to-child toxoplasmosis transmission. Out of the 12 interviews performed, we did not identify any diagnosed congenital toxoplasmosis in newborns.

In cohorts of women who have been screened routinely during pregnancy and treated accordingly, once the primary infection was diagnosed, the mother-to-child transmission (MTCT) rate was <5% after an acute primary maternal infection very early in pregnancy, but MTCT rates were much higher with acute maternal infections acquired later in pregnancy (15%, 44%, and 71% after maternal seroconversions at 13, 26, and 37 weeks of gestation, respectively) [39]. In Italy, an overall MTCT rate of 32.9% was reported, with values of 17.6% in the case of seroconversion in the first trimester, 40.5% in the second trimester and 53.3% in the third [10]. Additionally, it was shown that maternal symptoms, such as lymphadenopathy, following seroconversion during pregnancy were associated with a higher risk of congenital infection [40]. The reported MTCT rate varied between countries, from 0.8% in Norway [41], 4.8% in Germany [42], and 56% in Brazil [43]. In the present study, all women were diagnosed during the first trimester of the pregnancy.

Although toxoplasmosis is a notifiable disease in Romania, with passive surveillance, no information is provided on reported cases by the National Institute of Health in the annual reports from 2008 to 2022 [44]. Currently, the rate of congenital infection in Romania is largely unknown; the few published studies in Romanian journals have been reviewed in 2014. The authors concluded that from the evidence presented in the reviewed studies, it was difficult to estimate the rate of congenital toxoplasmosis [45]. In 2013, the first isolation and genetic characterization of a *T. gondii* strain from a symptomatic human case of congenital toxoplasmosis in Romania was published for the first time [46].

As pregnancy progresses, several alterations in the maternal immune system facilitate fetoplacental development and prevent fetal rejection [47], which may contribute to the development of an environment that facilitates the escape of *T. gondii* from the immune response [48]. In most cases, a previous maternal infection protects against congenital transmission. However, as presented in a review by [8], hormonal-induced alteration of systemic immunity occurs in some pregnancies and, in a small number of cases, can result in disease reactivation and, occasionally, vertical transmission. For instance, in Brazil, vertical transmission associated with reactivation during pregnancy occurred in nine out of the 246 assessed newborns, all mothers being negative for HIV [43]. In addition, other cases have been reported in which pregnant women with immune suppression due to HIV infection have resulted in congenital toxoplasmosis [49]. Due to the retrospective nature of our study, no information on associated diseases and possible immunosuppression in our patients was collected.

### 4.5. Other Considerations on Serological Profiles in the Investigated Patients

Persistent IgM serological profile during the same pregnancy, without IgG seroconversion, was described in two patients, of which one presented twice, while the other was tested four times during the same pregnancy and also in one patient in two consecutive pregnancies. IgM antibodies are not an accurate marker for discriminating between acute and late infection [37]. When IgM appears without specific IgG, further testing is required to diagnose recent infection because of the possibility of non-specific IgM [15]. IgM may persist months or even years after primary infection, and interpretation of IgM-positive results in the absence of IgG-positive antibodies as acute infection represents misdiagnosis and possibly wrong decision on pregnancy termination [15]. Though in the data sheets of the employed kits, there is no mention of anti-*Toxoplasma* antibodies cross-reactivity with Rubella and Cytomegalovirus, some cross-reactions between antibodies are inevitable in immunoassays and have been described in other studies [50,51]. The clinicians should carefully evaluate patients with TORCH IgM multipositive results to rule out potential false positives. Though we have evaluated seasonality in IgM-positive tests, no seasonal influence on *Toxoplasma* IgM-positive rates was observed, similar to a retrospective study performed on 10,669 women before pregnancy [51].

IgG antibodies appear after two weeks of infection and peak at three months, remaining at a plateau level for six months, and then slowly decrease to lower levels until the end of the infected subject’s life due to the persistence of latent cysts [52]. Although theoretically, IgG antibodies persist during the entire life following acute infection, our data show a decrease from positive to negative or borderline results in 34 patients rescreened during the second pregnancy. Interpretation of the *Toxoplasma* serological results depends on the sensitivity of laboratory tests used in practice [53], explaining possibly discordant results in case of low levels of IgG antibody titers that might be detected and reported as positive with one laboratory test and negative with another [54]. Due to the employment of numerous ELISA kits and automatic systems, no comparisons of antibody titers could be performed in our study. On the other hand, a decrease in IgG antibody titers in time might also be the explanation. That data also suggests that protection from infection might be present even in some patients with IgG negative results (negative due to a decrease in the titer of antibodies), as the importance of adaptive immune responses for resistance to *T. gondii* during human infection is demonstrated [55].

A false-positive IgG result during pregnancy can lead to a misdiagnosis of past infection and to stopping preventive measures. IgG false positivity for *T. gondii* due to past contact with parasites genetically close to *T. gondii*, such as *Neospora caninum,* has been described in humans and animals [56]. Data on the seroprevalence of *N. caninum* in dogs [57], dairy cattle [58], and dairy goats [59] are available from Romania but not in humans.

Persistent IgM and IgG positive serological profile in different pregnancies was documented in 11 patients. Interpretation of IgM and IgG serological profile as acute infection in the absence of avidity testing for IgG antibodies is often a cause of misdiagnosis and incorrect decision for pregnancy termination [36]. Our results sustain previously published data that IgM antibodies may persist for years after acute infection [37]. Even IgG low avidity results persisting for up to 5 years were documented [41], reinforcing the limits of serology and the need for a complex evaluation, including amniocentesis and PCR testing for *T. gondii* in suspected ATI in pregnancy [15].

There is currently evidence that hormones affect the course of toxoplasmosis in humans and mice [60]. A review by [61] on the most frequent parasitic infections during pregnancy suggests that biological factors produced during pregnancy regulate the immune response and may affect the establishment and reproduction of parasites by affecting the specific host-parasite immune response or serving the parasite as direct and positive growth factors. A review of eight studies in humans and 22 in animals and cell cultures tried to evaluate the relationship between hormones and infection caused by *T. gondii* but did not find comparable data [62]. It was recently shown that progesterone, a critical hormone that supports pregnancy, significantly inhibited the invasion and proliferation of tachyzoites and identified a *T. gondii* progesterone membrane receptor protein that proves to be an essential link between *T. gondii* and progesterone [63]. Still, further studies are required to determine the mechanism of hormone action in the *T. gondii* infectious process.

### 4.6. Strengths and Limitations

Our study strengths are represented by its large sample size, bringing information, as far as we know, on the largest number of pregnant women in Romania screened for *T. gondii* infection in a cohort study over a 15-year interval. Our study brings new data on *Toxoplasma* seroprevalence in Romania.

The retrospective nature of our study explains the absence of some data meaning for collection. A limitation is represented by the absence of information in the electronic files of pregnancy week at the first screening. Although our clinical protocol recommends testing in the first 8–10 weeks of pregnancy, late presentation is not excluded. Testing after the first trimester makes difficult the interpretation of serological results, and high avidity for IgG antibodies in the second or third trimester does not exclude ATI in early pregnancy.

## 5. Conclusions

The seroprevalence of anti-*Toxoplasma* IgG antibodies in pregnant women increased with patients’ age and decreased in time intervals during the 15-year study period. Although a high percentage of women included in our study were at risk for ATI during the pregnancy (70.88%), only a low percentage (0.9%) presented for rescreening during the same pregnancy. Our study proves poor compliance with the screening program in the Romanian population. ATI in pregnancy was identified as possible in 44 patients (0.16% of the total cohort). Investigation by telephone interview could not identify any congenital toxoplasmosis in children born from mothers with possible ATI. Further analysis of the cost-effectiveness of antenatal toxoplasmosis screening in Romania should be performed.

## Figures and Tables

**Table 1:** Demographic characteristics of the screened patients.

**Age**	Mean (Min–Max)	29.64 ± 4.82 (13–54)
	Median	29
**County of residence**	Cluj county (*n*; %)	23,239; 85.53%
	Other counties (*n*; %)	3930; 14.47%
**Environment**	Rural (*n*; %)	19,304; 71.05%
	Urban (*n*; %)	7865; 28.95%

**Table 2:** Screening results according to age group.

Age Group	n	%	IgM			IgG			IgM and IgG Negative		
			+	%	95% CI	+	%	95% CI	n	%	95% CI
[13.0–14.9]	12	0.04	0	0	0–26.46	3	25	5.49–57.19	9	75	42.81–94.51
[15.0–19.9]	529	1.67	5	0.95	0.40–2.19	166	31.38	27.57–35.47	360	68.05	63.96–71.88
[20.0–24.9]	3656	11.55	34	0.93	0.67–1.30	1067	29.19	27.74–30.69	2566	70.19	68.68–71.65
[25.0–29.9]	11,808	37.3	116	0.98	0.82–1.18	3221	27.28	26.49–28.09	8477	71.79	70.97–72.59
[30.0–34.9]	10,562	33.36	112	1.06	0.88–1.27	2937	27.81	26.96–28.67	7521	71.21	70.34–72.06
[35.0–39.9]	4293	13.56	48	1.12	0.84–1.48	1385	32.26	30.88–33.68	2838	66.11	64.68–67.51
[40.0–44.9]	768	2.43	9	1.17	0.62–2.21	290	37.76	34.40–41.24	465	60.55	57.05–63.94
[45.0–49.9]	28	0.09	0	0	0–12.34	13	46.43	27.51–66.13	14	50	30.65–69.35
[50+]	2	0.01	0	0	0–84.19	1	50	0.51–71.64	1	50	0.51–71.64
Total	31,658	100	324	1.02	0.92–1.14	9083	28.69	28.20–29.12	22,251	70.29	69.78–70.79

**Table 3:** Anti-*Toxoplasma* IgG antibodies seropositivity, expressed in five-year intervals.

Period	Positive Tests (%)		Positive Women (%)	
I (2008–2012)	30.04%	χ^2^ = 17.3; d.f. = 2; *p* = 0.0002	30.09%	χ^2^ = 8.4; d.f. = 2; *p* = 0.014
II (2013–2017)	29.23%		30.01%	
III (2018–2022)	27.39%		28.32%	

**Table 4:** The crosstabulation of IgM and IgG screening test results.

	IgG							Total	
		Negative		Borderline		Positive			
**IgM**	**negative**	22,251	70.29%	302	0.95%	8686	27.44%	31,239	98.68%
	**borderline**	5	0.02%	0	0%	90	0.28%	95	0.3%
	**positive**	17	0.05%	0	0%	307	0.97%	324	1.02%
**Total**		22,273	70.36%	302	0.95%	9083	28.69%	31,658	100%

## Data Availability

The complete dataset used and analyzed during the current study is available from the corresponding author upon reasonable request.

## Supplementary Materials

The following supporting information can be downloaded at https://www.mdpi.com/article/10.3390/microorganisms11092189/s1, Supplementary Materials: questionnaire.
