# Supplementary material for: Toxoplasmosis Screening during Pregnancy in a Romanian Infectious Diseases Tertiary Center: Results of a 15 Years Follow-Up Program
Source: Microorganisms. 2023 Aug 30;11(9):2189. doi: 10.3390/microorganisms11092189 (PMC10537741; doi:10.3390/microorganisms11092189)
Supplement: Supplementary file 1 [file microorganisms-11-02189-s001.zip › microorganisms-2545901-supplementary.pdf]

Patient name________________________

Patient age__________

Child's current age__________

1. Age of pregnancy when *Toxoplasma gondii* infection was diagnosed?

Week________________

2. Did you follow any treatment? □ yes □ no

If yes, with: spiramicyn (rovamicyn)? □ yes □ no

If yes, with: pyrimethamine + sulfadiazine □ yes □ no

Until the end of the pregnancy? □ yes

□ no, for how long? ____________________________

3. Did you give birth to the baby? □ yes

□ no : □ spontaneous abortion □ induced abortion

4. Amniocentesis for *Toxoplasma* performed? □ yes □ no

5. Was the birth on term? □ yes □ no

If not, in which week? _____________

6. Baby's weight at birth? ____________

Child's current weight?____________

7. APGAR score at birth? _____________

8. Was there a transfontanel ultrasound performed on the baby? □ yes □ no

9. Was there an ophthalmological exam performed? □ yes □ no

10. Was there an auditory exam performed? □ yes □ no

11. Did the child receive any treatment for toxoplasmosis? □ yes □ no

If yes, with what? ______________________

the duration of treatment? _________

12. Was the child tested for toxoplasmosis during its first year of life? □ yes □ no

If yes, frequency? ______________ and results _________________________

13. Does the child have visual impairment? □ yes □ no

If yes, what?___________________________________

Ophthalmological diagnosis______________________

14. Does the child have any heart problems? □ yes □ no

If yes, diagnosis_____________________________

15. Does the child have any neurological problems? □ yes □ no

If yes, what?________________________________

Neurological diagnosis_______________________

Has/had hydrocephalus? □ yes □ no

16. Does the child have any psychiatric problems?

If yes, what? ____________________________

Psychiatric diagnosis______________________

17. Does the child have any liver problems? □ yes □ no

If yes, diagnosis___________________________

18. Disease diagnoses from birth to present?

____

____

____

____

____
